# A framework for stimulating economic investments to prevent emerging diseases

**DOI:** 10.2471/BLT.17.199547

**Published:** 2017-11-14

**Authors:** Daniel L Schar, Gavin Mark Yamey, Catherine C Machalaba, William B Karesh

**Affiliations:** a United States Agency for International Development, Regional Development Mission for Asia, Athenee Tower, 25th Floor, 63 Wireless Road, Lumpini, Patumwan, 10330, Bangkok, Thailand.; b Duke Global Health Institute, Duke University, Durham, United States of America (USA).; c EcoHealth Alliance, New York, USA.

The increase in both the number of events and diversity of emerging infectious diseases is threatening public and animal health.^1^ Over the last decade alone, the global community has experienced the repeated burden of emerging diseases, from pandemic influenza to Ebola and Zika virus epidemics. Other pathogens, notably influenza A (H7N9), Middle East respiratory syndrome coronavirus and Nipah virus, have demonstrated their capacity to become global threats. In parallel, the increasing incidence of multidrug-resistant pathogens has become a pressing global health threat that challenges both the human and animal health sectors. The prospect of a post-antibiotic era prompted a high-level consultation on antimicrobial resistance at the 2016 United Nations General Assembly. It was only the fourth such occasion Member States convened and issued a declaration associated with a developing health crisis.^2^

Infectious disease emergence and multidrug-resistant pathogens are among this century’s defining global health challenges. The magnitude of their present and potential impact is sobering. In addition to the disease burden and social impact on families and communities, economic losses due to epidemics and pandemics are often more significant than the direct immediate and longer-term medical expenses. The World Bank estimates the global cost of the 2003 severe acute respiratory syndrome pandemic at 30 billion United States dollars (US$).^3^ The 2013–2016 Ebola virus disease outbreak was associated with US$ 2.2 billion in lost economic growth for Guinea, Liberia and Sierra Leone alone.^4^ Total costs attributable to both income loss and premature mortality from a moderately severe influenza pandemic are projected at US$ 570 billion annually, which is within the range of the annual cost associated with global climate change.^5^ Without intervention, the cumulative economic impact of antimicrobial resistance through 2050 is anticipated to exceed US$ 100 trillion, two-thirds of which would be in low- and middle-income countries, substantially more than current annual global economic output.^6^

Increasingly, the evidence base suggests that the accelerated rate at which infectious diseases of pandemic potential and antimicrobial resistance are emerging is strongly correlated with anthropogenic change on the planet.^7^ Rapid population growth and demographic shifts, coupled with soaring demand for animal-sourced nutrition, changes in food-production systems, global travel and trade, and an increasing remodelling of our natural landscapes, are opening new pathways to emerging and re-emerging diseases.

The World Bank and health and development institutions have made the case for investing in proactive, preventive measures that directly address these drivers, and for enhancing capacities that can contribute to averting the worst of their consequences. A yearly investment of US$ 1.9–3.4 billion to strengthen animal and human health systems would yield an estimated global public benefit of over US$ 30 billion annually, because it would avoid the economic damages associated with pandemics.^3^ High return on investment is expected even if only some pandemics are prevented. Strengthened multidisciplinary national capacities would bring additional benefits by improving prevention and control of endemic disease in human and animal populations. This improvement would initiate a cascade effect that would be expected to include lower rates of infectious disease-associated morbidity, reduced antimicrobial demand and ultimately, a scaling back of pressures fostering drug resistance.

Yet, despite the pattern of costly responses and a compelling investment case for prevention, global postures remain primarily response-driven and reactive to a dynamic and volatile emerging disease landscape. New epidemics are often met with an emergency response, after-action reviews and a promise to rethink prevention. So what can we learn from the failure to implement prevention and preparedness plans built on the premise that such events are avoidable?

Both cost–benefit and return on investment analyses have long been used for prioritizing public health resources, however, the use of these analyses in the complex human–animal–ecosystem health context is at an early stage. Where the analyses have been performed, results have not always gained support from budget decision-makers, limiting their translation into policies. In part, this resistance can be attributed to the dual constraints of competing priorities for human and veterinary health systems funding, and to the difficulty in mobilizing sufficient resources to fund infrastructure and risk mitigation efforts. Furthermore, there is limited investment in evidence-based animal reservoir surveillance and research of wildlife and environmental factors associated with emergence of infectious diseases. This limitation has frequently resulted in a general lack of awareness among policy-makers of upstream prevention opportunities, indirectly adding to resistance in adjusting priorities. Considering the broad array of factors contributing to the emergence of infectious disease, bridging inequitable distribution of costs and benefits across sectors and stakeholders also remains an obstacle.

A strategy for addressing these challenges is needed. We present here five interrelated pillars that constitute a framework to institutionalize investment-driven approaches to emerging infectious disease risk mitigation and to catalyse the transition to actionable prevention efforts.

First, we must further strengthen the evidence base demonstrating under what conditions investments in proactive and preventive disease mitigation approaches are fiscally prudent. The custodians of public health resources will demand conclusive evidence that redirecting finances into pre-empting future emergence events will, in fact, produce attractive returns on investment. This evidence must capture and quantify the cost of inaction. It will be important to account for previously hidden losses, reduced trade revenue, financial market shocks, social order disruption, premature mortality, increased all-cause morbidity and lost wages, stemming from epidemics and pandemics driven by business-as-usual practices, to ensure decisions are made on an equitable-comparison basis.^8^ Even in the pre-epidemic stage, as with animal-origin influenzas, livestock depopulation, lost productivity and follow-on effects impacting agricultural livelihoods and food security can pose a substantial economic burden.

Second, recognizing that a robust evidence base is necessary but not sufficient, economically informed, tested and validated innovations should be scaled. Land-use planning, for example, should account for the economic impact of disease emergence from a disrupted landscape. Only then will the disease-regulatory role of ecosystems be fairly valued and incorporated into payment for environmental services frameworks, similar to carbon sequestration and watershed conservation valuations.^9^

The challenge of transcending inequitable cost and benefit distribution is partially rooted in the failure to employ such economic analyses in directing policy and planning. Revenue that accrues to one sector, natural resource extractive industries, for example, is frequently offset by an economic burden in other sectors, including trade and commerce, health systems, communities and individuals. Solutions that employ a whole-of-society approach in equitably managing the risk and distributing benefits may provide a road map to address these inequities.

Third, a set of structures that incentivize investments in risk mitigation should be promoted. Global, regional and national level financing platforms, should, as a precondition of funding, require a re-balance towards a targeted strengthening of health systems. This re-balance should focus on surveillance, a multidisciplinary health workforce and health information systems essential for early detection and rapid outbreak response. More recently, the incorporation of disease emergence risk profiles into macro-economic analyses and bond ratings has been suggested as a potential tool offering a favourable investment environment in exchange for achievements in risk mitigation.^10^

Risk avoidance and risk transference models, including newly developed pandemic insurance structures powered by catastrophe modelling, can play a meaningful role in tilting the balance towards risk mitigation by discounting premiums for maintaining benchmark prevention capacities.^11^ Such benchmark capacities, could, for example, be dependent on achieving enhanced immunization coverage for vaccine-preventable diseases in humans and domestic animals, which would help reduce the overuse and inappropriate use of antimicrobials.

Fourth, funds for this transition must be mobilized. Official development assistance for health that targets strengthened prevention and control of cross-border infectious disease remains short of the levels required to be fully functional.^12^ Additional resources will need to be secured. Rethinking policy and regulatory options may yield solutions that generate additional revenue and incentivize risk mitigation, while penalizing business-as-usual practices that result in negative health outcomes. Options may include policies aimed at influencing industry practices, such as tax structures and emerging disease avoidance credits. The private sector has an important role here, and increasingly, investor-led coalitions are demanding risk mitigation practices as a precondition for investment.^13^

Finally, it is imperative that we collectively commit to treating emerging infectious disease prevention as a global public good given the inherently transboundary nature of infectious diseases and the non-rival, non-excludable benefits of their avoidance. Sustained, high-level advocacy, including through such channels as the Global Health Security Agenda,^14^ the Group of 7 and G20, are essential if we are to secure commitments to sound, well considered investments in our collective future prosperity. In parallel, economic analysis and incentives must also be delivered at country level, through national One Health platforms, for example, to make national health security plans actionable by multiple actors, including ministries of finance that can work across sectors and optimize budget allocations to improve preventive capacities.

Embedding this five-pillar approach into multidisciplinary One Health platforms presents an opportunity to apply the framework where the required constituencies and partnerships are present. A locally contextualized approach will yield insight into the validity of catalysing investments in prevention at the primary level of vested authority: national and subnational levels.

Economic principles should serve as the foundation for prioritizing preventive approaches. Ultimately, such approaches, built upon an evidence-based investment case, could shift the existing paradigm away from infectious disease emergence as inevitability, and towards avoidance. The transition to disease preventive practices will also deliver benefits extending across the sustainable development goals of the *2030 agenda for sustainable development*,^15^ from ending poverty and hunger to advancing environmental sustainability and economic growth.
